# A Nature-Inspired Betalainic Probe for Live-Cell Imaging of *Plasmodium*-Infected Erythrocytes

**DOI:** 10.1371/journal.pone.0053874

**Published:** 2013-01-16

**Authors:** Letícia Christina Pires Gonçalves, Renata Rosito Tonelli, Piero Bagnaresi, Renato Arruda Mortara, Antonio Gilberto Ferreira, Erick Leite Bastos

**Affiliations:** 1 Centro de Ciências Naturais e Humanas, Universidade Federal do ABC, Santo André, Sao Paulo, Brazil; 2 Departamento de Ciências Biológicas, Universidade Federal de São Paulo, Diadema, Sao Paulo, Brazil; 3 Departamento de Biofísica, Universidade Federal de São Paulo, São Paulo, Brazil; 4 Departamento de Microbiologia, Imunobiologia e Parasitologia, Escola Paulista de Medicina, Universidade Federal de São Paulo, São Paulo, Brazil; 5 Departamento de Química, Universidade Federal de São Carlos, São Carlos, Sao Paulo, Brazil; 6 Departamento de Química Fundamental, Instituto de Química, Universidade de São Paulo, São Paulo, Brazil; Julius-Maximilians-University Würzburg, Germany

## Abstract

A model betalainic dye was semisynthesized from betanin, the magenta pigment of the red beet, and was effective for live-cell imaging of *Plasmodium*-infected red blood cells. This water-soluble fluorescent probe is photostable, excitable in the visible region and cell membrane-permeable, and its photophysical properties are not notably pH-sensitive. Fluorescence imaging microscopy of erythrocytes infected with *Plasmodium falciparum*, a causative agent of malaria in humans, showed that only the parasite was stained. Z-stacking analysis suggested that the probe accumulates proximal to the nucleus of the parasite. Indicaxanthin, one of the natural fluorescent betalains found in the petals of certain flowers, did not stain the parasite or the red blood cell.

## Introduction

Betalains are water-soluble pigments responsible for the visible fluorescence of flowering succulent plants. [1] The biosynthesis of betalains is based on the oxidative cleavage of 3,4-dihydroxyphenylalanine (DOPA) in the presence of DOPA 4,5-dioxygenase, which is encoded by the *BvDODA1* gene [2], followed by a spontaneous aldimine coupling between the resulting betalamic acid (**HBt**) and amines or amino acids. [3] Natural betalains are non-toxic antioxidants known to bind to biological membranes and interact with human lipoproteins, most likely due to their conjugated amphiphilic chemical structures (Scheme S1). [4] Extracts of plants pigmented with betalains have been used in popular medicine for the treatment of a variety of diseases including malaria, [5] a severe infectious disease responsible for millions of deaths each year worldwide. [6].

*Plasmodium* species are the causative agents of malaria. These obligate, intracellular organisms have a complex life cycle and switch between a mosquito vector and a vertebrate host. [7] *Plasmodium* spp. merozoites infect erythrocytes and undergo a differentiation process that starts with the ring stage, followed by the trophozoite stage and finally the schizont stage. Differentiation inside the erythrocyte occurs within the parasitophorous vacuole (PV), a vacuolar membrane that surrounds the intracellular parasite. The sequential pathway for the entry of exogenous nutrients required for parasite survival and multiplication includes crossing the red blood cell membrane (RBCM), the parasitophorous vacuole membrane and the parasite plasma membrane. [8] An essential aspect of the infection process is the remodeling of the RBCM and its protein constituents to permit a higher flux of nutrients and waste products into or away from the intracellular parasite. [9] A single type of broad-specificity channel, variously called the new permeation pathway (NPP), the nutrient channel, or the plasmodial surface anion channel, is responsible for the increased permeability of low molecular weight solutes, both charged and uncharged, with a strong preference for anions. [10].

Inspired by the biological role of betalains, we sought to create a new small, tunable and water-soluble molecular framework for dyes suitable for live-cell fluorescence imaging. Due to the presence of at least two carboxyl groups in the betalain framework, such dyes are negatively charged at near-neutral pH and favor cell permeation through anion channels, such as the NPP. The functional group attached to the betalainic moiety modulates the amphiphilic character of the dye and influences its electronic properties. Therefore, we prepared the artificial coumarinic betalain **BtC** via aldimine coupling of the fluorescent hydrophobic chromophore 7-amino-4-methylcoumarin (C120) to **HBt**. This dye was used for the identification of erythrocytes infected by *Plasmodium* spp. using fluorescence imaging.

## Materials and Methods

### Ethics Statement

This study was carried out in strict accordance with the recommendations of the Council for International Organizations of Medical Sciences (*International Guiding Principles for Biomedical Research Involving Animals*, CIOMS, 1985). The protocol was approved by the Committee on the Ethics in Research of the Federal University of São Paulo (Permit Number: 1364/09). All surgery was performed under anesthesia, and all efforts were made to minimize animal suffering.

### I. Semisynthesis

#### I.1 General

All chemicals were purchased from Sigma-Aldrich and used without further purification, except where noted. The NMR spectra were recorded using a Bruker Avance (9.4 T, 400 MHz) or Bruker Avance III (14.1 T, 600 MHz) spectrometer. Chemical shifts are reported as δ values (ppm) referenced to the solvent residual signals: D_2_O, δ_H_ = 4.63 (*s*) ppm, TFE δ_H_ = 3.88 (*m*) and 5.02 (*s*) ppm. High-resolution mass spectra were obtained using a Bruker MicroTOF-QII mass spectrometer. Flash column chromatography was performed using 70–230 mesh silica gel. Absorption spectra were recorded in the visible region of the electromagnetic spectra (380–780 nm) at 25±1°C using a Varian Cary 50 Bio spectrophotometer equipped with a Peltier thermostated cell holder. Fluorescence spectroscopy studies were performed using a Varian Cary Eclipse spectrometer connected to a thermostatic bath. The excitation and emission spectra were recorded at 25°C.

#### I.2 Purification of betanin

Beetroots (*Beta vulgaris* subsp. *vulgaris* var. *vulgaris,* 0.5 kg) were peeled, sliced and homogenized in a centrifugal juice extractor (Phillips–Walita, RI1858) at the maximum speed. The juice was centrifuged (1370×g, 30 min, 25°C) and filtered (Whatman qualitative filter paper, grade 4), and the supernatant was stored at –20°C and used within 5 days. The betanin/isobetanin mixture was purified from beetroot juice by reversed-phase column chromatography (silica gel 90 C_18_-RP; 20 g; conditioned and eluted with deionized water at a flow rate of 0.3 mL min^–1^). The concentration of betanin was determined by assuming a molar absorption coefficient (*ε*) of 6.5×10^4^L mol^–1^cm^–1^ at 536 nm. [11].

#### I.3 Betalamic acid

Betalamic acid was obtained by the alkaline hydrolysis of betanin, as previously described. [12].

**UV-Vis:** λ_abs_^max^ 430 nm (water, pH 11), *ε* ^430 nm^ = 2.65×10^4^ L mol^–1^ cm^–1^ (water, pH 11.4); **Fl:** λ_Fl_^max^ 500 nm (water, pH 11, λ_exc_ 420 nm).

#### I.4 Indicaxanthin (BtP)

L-Proline (100 equiv.) was added to an aqueous solution of betalamic acid (0.5–1.0 mL, pH 10), and the resulting solution was stirred at room temperature (RT). The reaction was spectrophotometrically monitored by the depletion of the **HBt** absorption band at 430 nm and the concomitant appearance of the **BtP** band at 485 nm. After completion (ca. 30 min), the solution was cooled (0°C), and HOAc (*conc.*) was slowly added until the solution reached pH 5. The resulting solution was then stirred at RT for 1 h. The product was purified by reversed-phase column chromatography (C18 silica gel, water as eluent), and the fractions containing **BtP** were combined and lyophilized. The resulting orange powder was stored at −20°C, and the purity of the product was assessed by RP-HPLC analysis prior to use. In a typical run, 0.5 mg (2.5 µmol) of betalamic acid yielded 0.7 mg (2 µmol, 80%) of **BtP**.

**^1^H-NMR (400 MHz, D_2_O/TMSP-d4,** **according to Stintzing et al.** [**13**] **):** δ 8.34 (d, 1H, ^3^ *J_7_*_′,*8*′_ = 12.4 Hz, H-7′), 8.24 (d, 1H, ^3^ *J_7,8_* = 12.1 Hz, H-7), 6.20 (s, 1H, H-14), 6.16 (s, 1H, H-14), 6.08 (d, 1H, ^3^ *J_7,8_* = 12.1 Hz, H-8), 5.80 (d, 1H, ^3^ *J_7_*_′,*8*′_ = 12.4 Hz, H-8′), 4.47 (t, 2H, ^3^ *J_10,11_* = 6.6 Hz, overlapped H-11 and H-11′), 3.76–3.71 (m, 2H, H-5), 3.33 (dd, 2H, ^2^ *J_10a,10b_* = 17.2 Hz, ^3^ *J_10a,11_* = 6.6 Hz, H-10a), 3.11 (dd, 2H, ^2^ *J_10a,10b_* = 17.2 Hz, ^3^ *J_10b,11_* = 6.6 Hz, H-10b), 2.20–2.27 (m, 2H, overlapped H-3 and H-3′), 2.27–2.37 (m, 1H, H-3′), 2.43–2.54 (m, 1H, H-3′), 2.04–2.20 (m, 1H, H-4), 2.00–2.15 (m, 1H, H-4′).

**HRMS (** ***m/z*** **):** [M+H]^+^ calculated for C_14_H_17_N_2_O_6_^+^: 309.1087; observed: 309.1082 (dif.: 1.62 ppm).

**LC-ESI(+)-MS:** *R_t_* = 6.2 min, (*m*/*z*) [M+H]^+^ observed: 309.1.

**UV/Vis:** λ_max_ 485 nm (water), *ε* ^485 nm^ = 4.8×10^4^ L mol^–1^cm^–1^ (water) [14], [15].

**Fluorescence**: λ_max_^fl^ 520 nm (water, λ_exc_ 510 nm), Φ_Fl_ = 4.6×10^–3^ (water, pH = 6) *vs.* fluorescein.

**I.4 BtC.** 7-Amino-3-methylcoumarin (100 equiv.) was added to an aqueous solution of betalamic acid (0.7 mg (3.2 µmol) in 1.0 mL, pH 10), and the solution was submitted to ultrasonic irradiation for 5 min and stirred at RT for an additional 30 min. The reaction was spectrophotometrically monitored for the appearance of the **BtC** band at 520 nm. After completion, the solution was cooled (0°C), and HCl (*conc.*) was slowly added until the solution reached pH 3. The resulting solution was then stirred at RT in the dark for 2 hours. The product was purified via gel-permeation chromatography using lipophilic Sephadex LH-20 with water as the eluent. The fractions containing **BtC** were combined and lyophilized. The resulting purple powder was stored at −20°C, and the purity of the product was verified by RP-HPLC analysis prior to use. In a typical run, 0.7 mg (3.2 µmol) of betalamic acid yielded 0.4 mg (1 µmol, 31%) of **BtC**.

**^1^H-NMR** (600 MHz, D_2_O/TMSP-d4, Scheme S3): δ 8.46 (s, 1H, N-H), 8.08 (d, ^3^ *J_9,10_* = 12.4 Hz, 1H, H-10), 7.73 (d, ^3^ *J_13,14_* = 8.4 Hz, 1H, H-14), 7.17 (d, ^3^ *J_13,14_* = 8.4 Hz, 1H, H-13), 7.12 (bs, 1H, H-17), 6.27 (s, 1H, H-5), 6.23 (s, 1H, H-20), 6.07 (d, ^3^ *J_9,10_* = 12.4 Hz, 1H, H-9), 4.40 (bt, ^3^ *J_2,3a_* = 8.3 Hz, 1H, H-2), 3.30 (dd, ^2^ *J_3a,3b_* = 17.5 Hz, ^3^ *J_2,3a_* = 8.3 Hz, 1H, H-3a), 3.10 (dd, ^2^ *J_3a,3b_* = 17.5 Hz, ^3^ *J_2,3b_* = 8.3 Hz, 1H, H-3b), 2.43 (s, 3H, H-22).

**^1^H-NMR** (600 MHz, TFE+capillary D_2_O/TMSP-d4, Scheme S3): δ 8.66 (s, 1H, N-H), 8.44 (d, ^3^ *J_9,10_* = 12.8 Hz, 1H, H-10), 8.02 (d, ^3^ *J_13,14_* = 8.8 Hz, 1H, H-14), 7.55 (dd, ^3^ *J_13,14_* = 8.8 Hz,^ 4^ *J_13,17_* = 2.2 Hz, 1H, H-13), 7.44 (d, ^4^ *J_13,17_* = 2.2 Hz, 1H, H-17), 6.76 (s, 1H, H-5), 6.52 (d, ^3^ *J_9,10_* = 12.8 Hz, 1H, H-9), 6.50 (s, 1H, H-20), 4.50 (H-2, overlapped with TFE signal), 3.63 (dd, ^2^ *J_3a,3b_* = 17.7 Hz, ^3^ *J_2,3a_* = 7.2 Hz, 1H, H-3a), 3.34 (dd, ^2^ *J_3a,3b_* = 17.7 Hz, 1H, H-3b), 2.72 (s, 3H, H-22).

**^13^C-NMR** (125 MHz, D_2_O/TMSP-d4, Scheme S3): δ 147.7 (C-10), 129.4 (C-14), 116.2 (C-13), 114.1 (C-20), 110.4 (C-9), 108.4 (C-5), 105.7 (C-17), 56.6 (C-2), 20.7 (C-22).

**^13^C-NMR** (125 MHz, TFE+capillary D_2_O/TMSP-d4, Scheme S3): δ (ppm) 148.3 (C-10), 129.6 (C-14), 116.4 (C-13), 114.6 (C-20), 111.2 (C-9), 109.6 (C-5), 107.4 (C-17), 20.1 (C-22).

**LC-ESI(+)-MS:** *R_t_* = 5.6 min, (*m*/*z*) [M+H]^+^ observed, 369.1.

**HRMS (** ***m/z*** **):** [M+H]^+^ calculated for C_19_H_17_N_2_O_6_^+^, 369.1078; observed, 369.1082 (dif.: –1.08 ppm).

**UV/Vis:** λ_max_ 520 nm (water), *ε* ^520 nm^ = 6.6×10^4^ L mol^–1^cm^–1^ (water).

**Color:** CIE Lab D65/10°: L* = 90.4, a* = 25.9, b* = –3.9, C* = 26.2 and *h*° = 351.4.

**Fluorescence**: λ_max_^fl^ 570 nm (water, λ_exc_ 510 nm), Φ_Fl_ = 4.3×10^–3^ (water) *vs.* fluorescein.

### II. Spectrophotometric Methods

The **molar absorption coefficient (** ***ε*** **)** of **BtC** was determined using an end-point method by performing a set of betalain degradation experiments, as previously described. [16] Briefly, the alkaline hydrolysis of **BtC** (Britton-Robinson buffer, pH 9) was spectrophotometrically monitored for 30 min at 1-min intervals. The initial pigment concentration and *ε* of **BtC** were determined by comparing the absorption of the resulting betalamic acid solution with that of betanin solutions of known concentrations. The betalamic acid solutions were stable under the experimental conditions, and no appreciable change in the spectral properties could be detected after 30 min.

**Color analysis** was performed using the CIE L*a*b D-65/10° color space employing the *Color* software (v.3.1, Startek Technologies). L* indicates the lightness, and its value ranges from 0 (an ideal black object) to 100 (an ideal white object). In the CIE L*a*b* system, a* and b* are the chromaticity coordinates. A positive a* value indicates the red direction, and a negative a* value is the green direction; a positive b* value is the yellow direction, and a negative b* value is the blue direction. Two derived color parameters, the hue angle (*h*° = arctan2(b*,a*)×180/π) and the Chroma value (C* = (a* ^2^+ b* ^2^)^½^), were calculated.

The **fluorescence quantum yield (Φ_FL_)** was measured relative to fluorescein (NaOH_(aq)_ 0.1 mol L^–1^, Φ_FL_ = 0.79, EX 470–500 nm, EM 500–600 nm) with excitation at 475 nm. Fluorescence spectra of the standard and **BtC** were obtained under identical spectrometer conditions in triplicate and averaged (excitation at 475 nm, slits 2.5 nm (EX) and 5.0 nm (EM), photomultiplier power 600 V). The optical density at the peak was maintained below 0.1 to avoid inner-filter effects, and the integrated intensities of the emission spectra, corrected for differences in the indexes of refraction and concentration, were used to calculate the Φ_FL_ using Eq. 1,
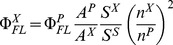
(1)where *A* is the absorbance at 475 nm, *n* is the refractive index of the solvent, *S* is the integrated area under the fluorescence spectrum and Φ_FL_ are the absolute quantum yields; the superscripts *X* and *P* refer to the sample and standard, respectively.

### III. Analytical Methods

#### Reversed-phase high-performance liquid chromatography (RP-HPLC) analysis

Analytical RP-HPLC separations and analyses were performed using a Waters 2695 Alliance system instrument with a UV-Vis detector (dual-wavelength, Waters 2489) and a Supelcosil LC-18 (5 µm particle size, L×I.D.: 150×46 mm, Supelco) C18 column. The *LC analysis conditions* were as follows: Solvent A, water with 0.1% v/v TFA; Solvent B, 60% v/v MeCN/water with 0.1% v/v TFA, linear gradient from 5% to 95% B in 20 min at 25°C, flow rate: 1 mL min^–1^, injection volume: 10 µL, spectrophotometric detection set at 254/536 nm for **Bn**, 484 nm for **BtP** and 520 nm for **BtC**. The analysis of **BtC** was performed in the absence of acid.

**HPLC-DAD-ESI(+)-MS/MS analysis** was performed using a Bruker Daltonics Esquire HCT ion trap mass spectrometer equipped with an electrospray source and coupled to a Shimadzu Prominence liquid chromatograph. The chromatograph was equipped with a Luna C18 column (3 µm particle size, L×I.D.: 150×2.0 mm, Phenomenex) maintained at 30°C and a PDA SPD-M20A detector. Nitrogen was used as the nebulizing (45 psi) and drying gas (6 L min^–1^, 300°C), and helium was used as the buffer gas (4×10^–6^ mbar). The capillary high voltage was set to 3,500 V. To avoid space-charge effects, the smart ion charge control (ICC) was set to the arbitrary value of 50,000.

**High-resolution mass spectrometric (HRMS) analysis** was performed using a Bruker MicroTOF-QII spectrometer with an electrospray ionization source with a capillary potential of 4.5 kV. Nitrogen was used as the nebulizing (5.8 psi) and drying gas (4 L min^–1^, 180°C). An internal calibration was performed using a sodium formate solution (1.0 mg mL^–1^) before and after each acquisition. The samples were introduced into the spectrometer by direct infusion at a constant flux of 3.0 µL min^–1^. The data were acquired and analyzed using the Bruker Compass software.

### IV. Experiments with Parasites

#### Parasite cultivation

*Plasmodium chabaudi* (clone AS) was maintained in BALB/C mice by weekly transfers from previously infected mice. Animals infected at the trophozoite stage (parasitaemia approximately 60%) were sacrificed, the blood was collected and leukocytes and platelets were removed from the whole blood by filtration through a powdered cellulose column (Whatman CF11). The trophozoite-infected erythrocytes were then washed three times by centrifugation (1,500×g, 5 min) in PBS. The parasites (10^7^ cells mL^–1^) were isolated by the lysis of the erythrocyte membranes with saponin in PBS (10 mg mL^–1^). After pelleting to remove the red blood cell membranes, the parasites were washed twice in PBS by centrifugation (2,000×g, 4°C). *Plasmodium falciparum* (3D7 strain) was cultured as previously described [17] in RPMI 1640 medium supplemented with 10% human serum in an atmosphere of 5% O_2_, 5% CO_2_ and 90% N_2_.

#### Epifluorescence microscopy

The parasitized erythrocytes were mounted wet on a glass-bottom petri dish (MatTek Corporation) treated with poly-L-lysine, incubated with **BtC** for 2 min, washed once with PBS and imaged within 10 min at 20 °C using a Zeiss Axio Observer Z1 epifluorescence microscope (Carl Zeiss MicroImaging, 38 HE GFP filter set, excitation band-pass 470/40, beam splitter FT 495, emission band-pass 525/50) with a 63×/1.25 oil-immersion objective, image acquisition time was 10,000 ms. Differential interference contrast (DIC) images were obtained using transmitted light and recorded using the Axio Vision software.

#### Confocal fluorescence microscopy

Cells were treated as described for the epifluorescence imaging and incubated for 2 min in the dark at 4°C with **BtC** (35 µmol L^–1^) and washed three times with PBS. The cells were subsequently stained with Hoechst 33258 dye (3.6 µmol L^–1^) for 2 min. The coverslips were mounted on slides using 4 µL of ProLong Gold (Invitrogen) and observed in a Leica SP5 TS confocal microscope with a 100×/1.44 oil-immersion objective; the Z series was acquired according to the sampling criteria built into the software for sequential imaging of the Hoechst 33258 dye, which stains the nucleus (blue, EX/EM = 350/470 nm) and the **BtC** (green, at EX/EM = 520/570 nm). All images (8-bit grayscale) were acquired with the same exposure time and sensibility. The raw images were processed with the ImageJ software.

### V. Computational Methods

Gaussian09 was used for all of the quantum-chemical calculations. [18] All of the structures were optimized in the gas phase at the cam-B3LYP/6-31+G(d,p) level. [19] Stationary points were characterized as minima based on vibrational analysis, and the coordinates of **BtC** in seven different protonation states are provided. The SMD parameterization of the IEFPCM was employed to model the effect of the solvent (water). Molecular descriptors of the betalains were estimated as the means of the Marvin Sketch 5.10.3 prediction program (ChemAxon, Budapest, Hungary). The LogD was predicted according to the method of Viswanadhan and collaborators. [20] The p*K*_a_ values of the carboxylic portions of **BtC** were obtained based on the partial charges, [21] and the values were compared to those determined by quantum mechanics using a thermodynamic cycle [22].

## Results and Discussion

Betalamic acid was prepared via the alkaline hydrolysis of betanin (**Bn**, purified from red beet juice [23]) and then coupled with C120 in water to yield **BtC**. The dye was purified by gel-permeation chromatography on lipophilic Sephadex LH-20 and subsequently characterized (File S1). The absorption and fluorescence spectra of **BtC** (λ^abs, W^ = 520 nm, λ^Fl, W^ = 570 nm, *h*° = 351 (D65/10°), ε^W^ = 66,000 L mol^–1^ cm^–1^) are red-shifted relative to that of C120 (λ^abs, MeOH^ = 343 nm, λ^Fl, MeOH^ = 445 nm, ε^MeOH = ^16,180 L mol^–1^ cm^–1^) [24] due to the extended conjugation of **BtC**. Furthermore, the Stokes shift of the betalain is smaller than that of C120 (*i.e.*, Δν_C120_ = 6683 cm^–1^ (102 nm) *vs.* Δν**_BtC_** = 1687 cm^–1^ (50 nm), Figure S1). The betalain **BtC** is water-soluble and excitable with visible light, two relevant improvements over the majority of coumarins used as fluorescent probes. However, **BtC** is several orders of magnitude less fluorescent in solution than is C120 (fluorescence quantum yield (Φ_FL_) of **BtC** in water = 4.3×10^–3^ *vs.* Φ_FL_^C120, MeOH^ = 0.51). The lack of a fluorescence signal from the uninfected erythrocytes is likely a result of the low quantum yield of **BtC**. However, a weak fluorescence signal is convenient for the quantification of the probe uptake and accumulation inside the parasite. [25] Finally, **BtC** is reasonably stable in aqueous media [half-life: 118±10 min at pH 7.0 (*k*_obs_ = 6.4±1.0×10^–3 ^min^–1^ (HPLC); 5.4±0.8×10^–3 ^min^–1^ (UV-Vis) (Figure S2), p*K*_h_ (hydrolysis constant) = 9.0 (Figure S3), half-life: 2 days in 60% v/v aqueous 2,2,2-trifluoroethanol [26]] and only weakly prone to photobleaching, as indicated by the emission profile under pulsed light irradiation at 520 nm in the presence or absence of infected erythrocytes (Figure S4).

**Figure 1:**
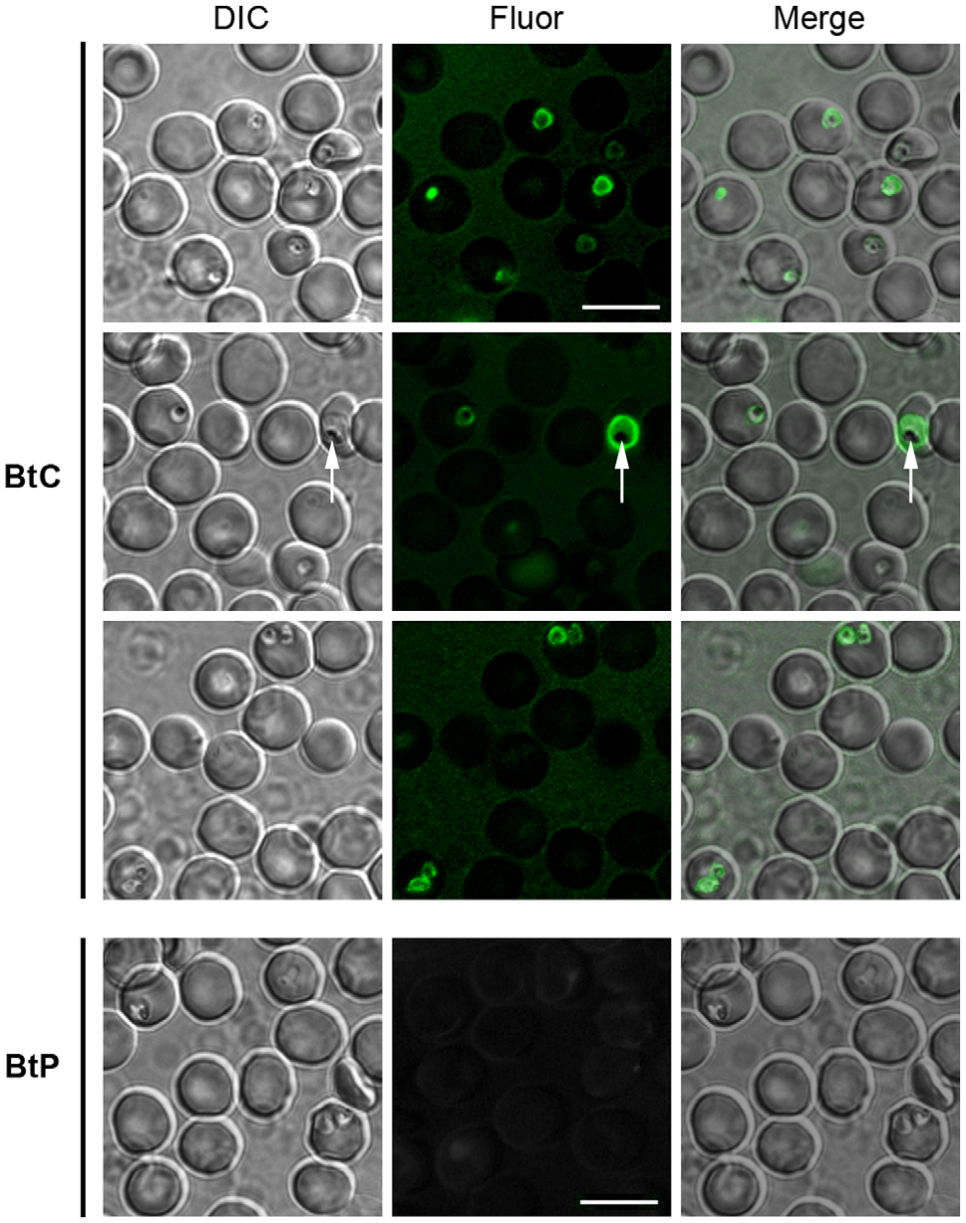
Labeling of live *P. falciparum*-infected erythrocytes with BtC. **BtP** was used as the betalain control. Panels show representative wide-field images of three independent experiments. The arrow indicates the non-fluorescent hemozoin crystal inside the DV of a mature trophozoite. [**BtC**] = 28 µmol L^–1^; [**BtP**] = 170 µmol L^–1^; incubation = 2 min at 25°C; bar = 10 µm.

To test the ability of **BtC** to label living *Plasmodium* spp.-infected red blood cells, parasitized erythrocytes were incubated with **BtC** and examined by fluorescence microscopy. Tests with *P. berghei*, *P. chabaudi* and *P. falciparum* yielded similar results; therefore, only the results for the labeling of *P. falciparum* are presented. As shown in Figure 1, **BtC** selectively accumulates within the infected cells at the point of parasite localization, even in doubly infected erythrocytes. Importantly, no stain was observed in uninfected cells or in infected-erythrocytes incubated with a control probe, indicaxanthin (**BtP**, Φ_FL_ = 4.6×10^–3^ at pH 6). In more mature stages of the intraerythrocytic developmental cycle, the accumulation of **BtC** is even more evident because fluorescence was commonly observed throughout almost the entire parasite (excluding the hemozoin crystals, which are most likely accumulated in the digestive vacuole (DV)). Furthermore, the fluorescence profile of the probe within the parasite is nearly identical to that in aqueous solution, indicating that the dye is not chemically modified upon uptake (Figures S4 and S5).

**Figure 2:**
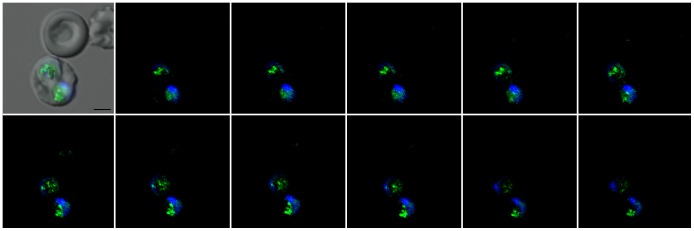
Z-Stack images (interval = 0.2 µm) of an erythrocyte infected with two parasites (*P. falciparum*). The first image is an overlay of the DIC image, the fluorescence signal from the **BtC** probe (pseudo-colored green) and the Hoechst dye (pseudo-colored blue). [**BtC**] = 35 µmol L^–1^; bar = 2 µm.

Confocal Z-stack sections of *P. falciparum-*infected erythrocytes revealed granular fluorescence in an unidentified compartment within the cytoplasm of the parasite (Figure 2 and Video S1). These results indicate that **BtC** may be taken up from the medium into the erythrocyte and then transferred to the parasite, where it accumulates within this intraparasitic compartment. Interestingly, the fixation of infected cell cultures with methanol, acetone or paraformaldehyde prior to or after incubation with **BtC** resulted in a loss of fluorescence (data not shown). The increase in cell membrane permeability caused by most fixing agents may favor the passive diffusion of the fluorescent probe and compromise the accumulation of the dye. [27].

Although the mechanism responsible for the vacuolar accumulation of **BtC** within *Plasmodium* remains undetermined, it is known that **Bn** and **BtP** are both incorporated into intact red blood cells. [28] The uptake of secondary metabolites is often coupled with a pH gradient between the vacuoles and the cytosol. The intracellular pH (pH_i_) of red blood cells, 7.33 [29], is higher than that of the parasite’s cytosol (pH_i_ = 7.1) [30], cytostomal vesicles (pH_i_ = 7) [31] or DV (pH_i_ = 5.4–5.5) [32]. **BtC** is expected to be fully protonated at pH <3 and has a double negative charge at pH 7.4 (Scheme S2). The experimental p*K*_aH_ of the imine moiety was determined to be 5.0±0.2, although the Φ_FL_ of **BtC** does not depend on the pH of the medium from pH 3 to 8 (Figure S3). Although **BtC** occurs in a more negative form in the cytoplasm than inside the acidic vacuoles, dye accumulation cannot be easily explained exclusively on this basis. Lipophilicity may also play a fundamental role in the vacuolar uptake of the betalainic dye. The theoretical distribution coefficient logD values of **BtP** are –5.5, –8.3 and –9.7 at pH 5.0, 6.5 and 7.4, respectively, which agrees with the reported experimental value of –7.25 at pH 6.0 [4]. For **BtC**, the values of logD are less negative, i.e., –1.8 (pH 5.0), –3.2 (pH 6.5) and –3.9 (pH 7.4), which suggests that the increased lipophilicity of the coumarinic betalain may favor the crossing of cellular membranes. However, active transport cannot be ruled out because the uptake of betaxanthins into the vacuoles of an artificial mini-protoplast system has been reported to be MgATP-dependent. [33].

The lack of genetic material in red blood cells allows the detection of malaria parasites in blood samples by light or fluorescence microscopy by using DNA/RNA staining methods. [34] Giemsa staining is frequently used to examine blood films and smears due to its high sensitivity and low cost. The drawbacks of this method include extensive cell manipulation, long-term requirement (45 min or even longer), and the occurrence of either artifacts and pigment dots. [35], [36] Staining with the **BtC** probe requires less than 5 min of incubation and allows live-cell imaging with minimal cell manipulation. Acridine orange is widely used for the fluorescent staining of parasitic DNA/RNA because it also circumvents some problems of the Giemsa method. However, despite the speed and versatility of the acridine orange staining, [35] this dye was found to be carcinogenic. [37], [38] Other organic fluorophores are available for functional parasite imaging, such as the calcium-sensitive dye Fura Red, which has been used to determine the accumulation of calcium ions in the parasite food vacuole. [39] Malaria parasites have also been selectively imaged with wide-field fluorescence microscopy using fluorescent proteins tags or quantum dots. [34] However, most of these methods are designed for the study of specific cellular processes and are often more complex and expensive than the **BtC** staining.

In conclusion, **BtC** belongs to a new class of fluorescent dyes that can be easily semisynthesized from an abundant, non-toxic and inexpensive natural pigment. These betalamic conjugates have tunable properties through variations of the imine substituent and improved water solubility and affinity to biomolecules relative to the precursor amines. Finally, the betalamic acid scaffold may be useful for the rational design of selective probes for other biological imaging applications, including fluorescent protein tagging.

## Supporting Information

Figure S1 **Normalized absorption (–) and fluorescence emission (····) spectra of C120 (black) and BtC (green) in PBS.**
(TIFF)

Figure S2 **Hydrolysis of BtC monitored by UV-Vis absorption spectroscopy or HPLC-DAD analysis.** (**A**) Changes in the absorption spectrum of **BtC** in aqueous buffer (pH = 7.0) over 66 h and (**B**) the corresponding kinetic profile; (**C**) Changes in the chromatographic profile of **BtC** over 19 h and (**D**) the corresponding kinetic profile of **HBt** (*red*, t_R_ = 1.2 min), **BtC** (*black*, t_R_ = 5.5 min), **C120** (*blue*, t_R_ = 11.6 min) and an unknown decomposition product (*green*, t_R_ = 2.4 min).
(TIFF)

Figure S3 (**A**) Effect of pH on the absorption and fluorescence spectra of **BtC,** and (**B**) dependence of the second-order absorption and emission maxima on the pH.
(TIFF)

Figure S4 **Fluorescence decay of BtC in water, in isolated** ***P. chabaudi*** **and in** ***P. chabaudi*** **-infected red blood cells.** Experiments with parasites were performed with an incubation – centrifugation – washing – resuspension sequence. [**BtC**] = 50 µmol L^–1^; EM 570 nm.
(TIFF)

Figure S5 **Fluorescence profiles of BtC in aqueous solution (control) and in suspension containing** ***P. chabaudi*** **-infected erythrocytes ([BtC] = 50 µmol L^–1^, 5 min incubation and washing (5×), EX 520 nm).**
(TIFF)

File S1 **NMR and HRMS spectra of BtP and BtC and cartesian coordinates of optimized structures.**
(DOCX)

Scheme S1 (**A**) Chemical structures of betanin (**Bn**) and indicaxanthin (**BtP**); (**B**) semisynthesis of **BtC**.
(TIFF)

Scheme S2 Acid–base equilibria of BtC.
(TIFF)

Scheme S3 Attribution of NMR signals of BtC; s: singlet, d: doublet, t: triplet, bt: broad triplet, bs: broad singlet, dd: double doublet, m: multiplet.
(TIFF)

Video S1 **3D Rotation of an erythrocyte infected with two parasites.** Staining: **BtC** (pseudo-colored green) and Hoechst dye (pseudo-colored blue).
(AVI)
