# Supplementary figures and images for: A Nature-Inspired Betalainic Probe for Live-Cell Imaging of Plasmodium-Infected Erythrocytes
Source: PLoS One. 2013 Jan 16;8(1):e53874. doi: 10.1371/journal.pone.0053874 (PMC3547039; doi:10.1371/journal.pone.0053874)

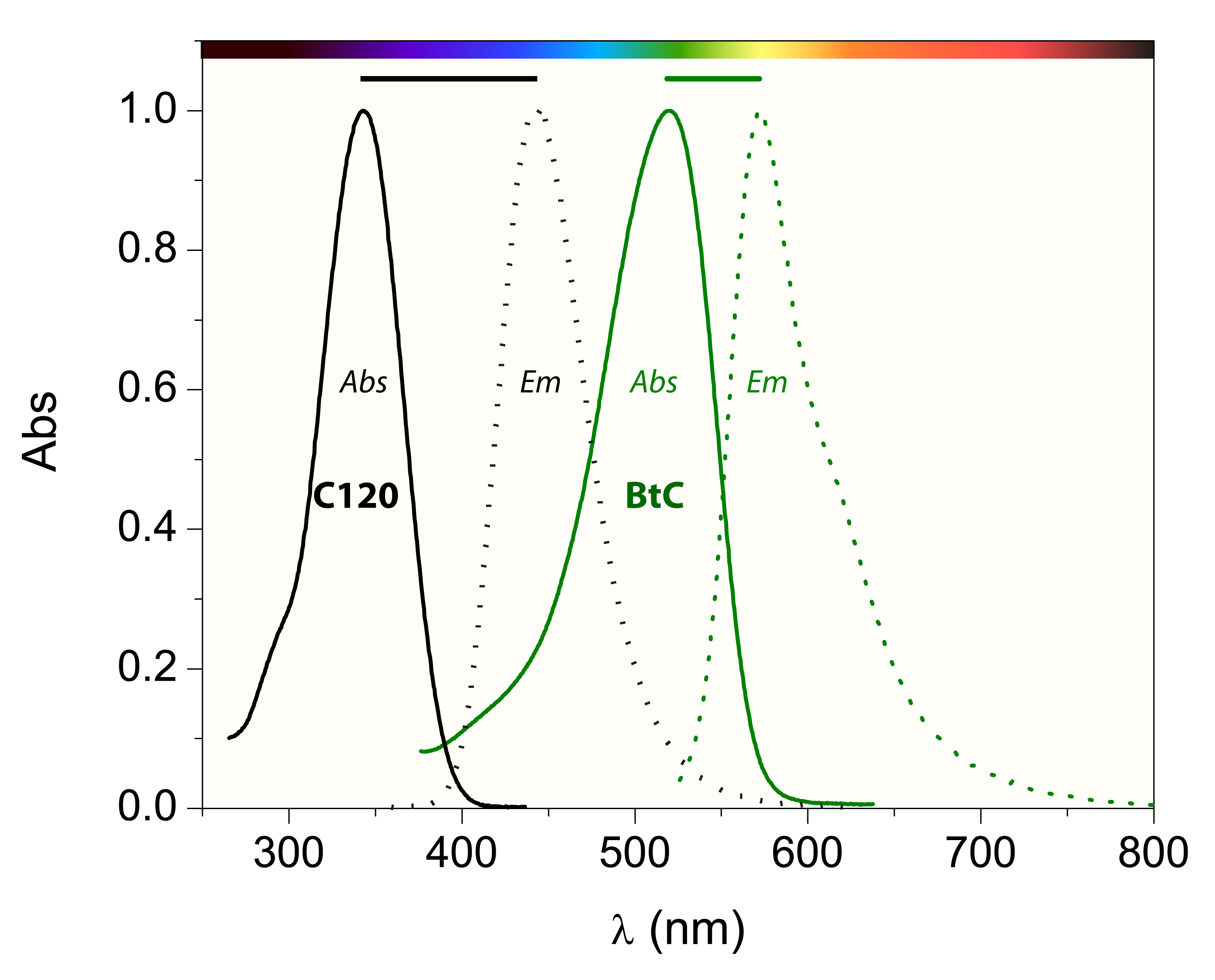

Supplement: Figure S1 — Normalized absorption (–) and fluorescence emission (····) spectra of C120 (black) and BtC (green) in PBS. (TIFF) [file pone.0053874.s001.tif]

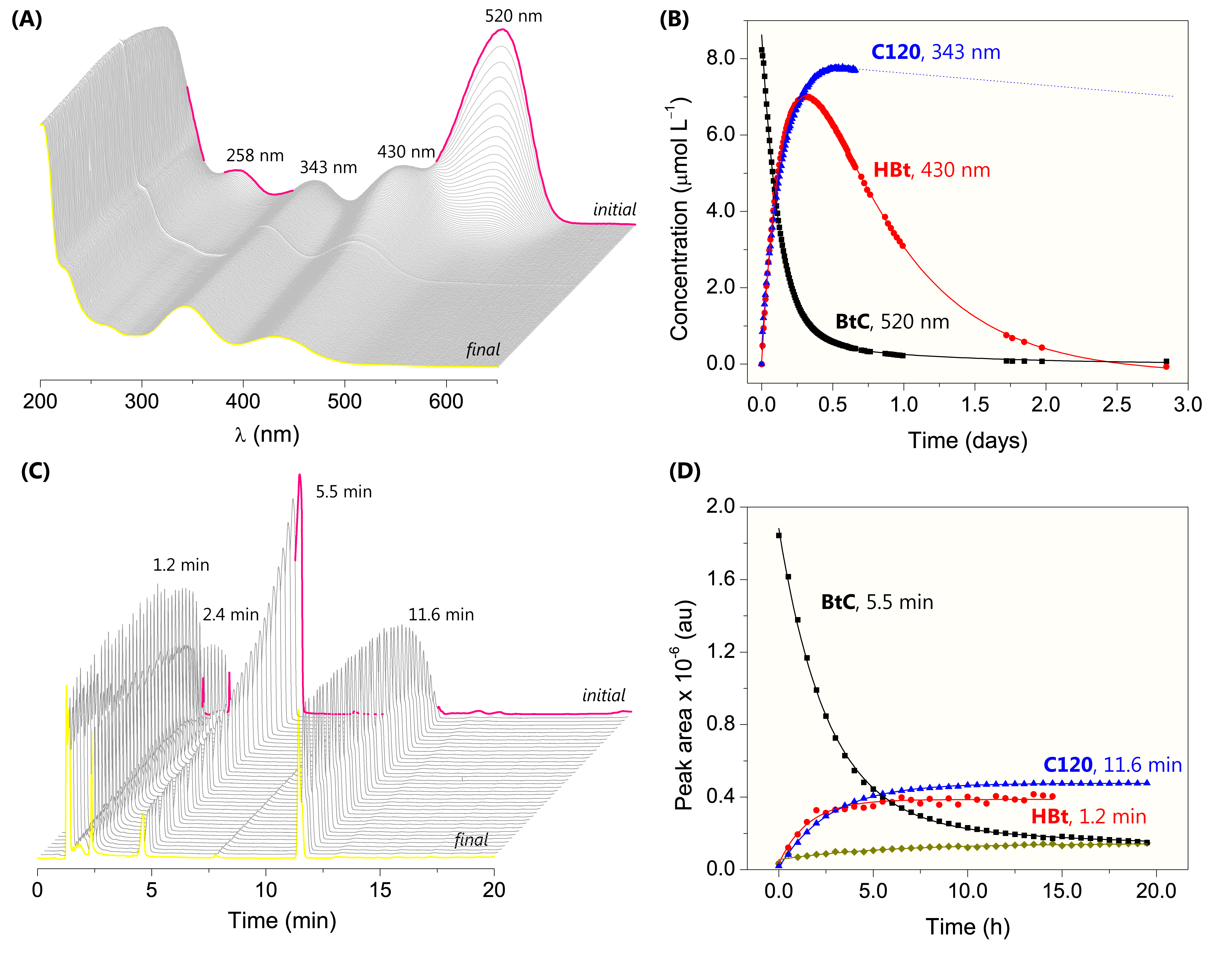

Supplement: Figure S2 — Hydrolysis of BtC monitored by UV-Vis absorption spectroscopy or HPLC-DAD analysis. (A) Changes in the absorption spectrum of BtC in aqueous buffer (pH = 7.0) over 66 h and (B) the corresponding kinetic profile; (C) Changes in the chromatographic profile of BtC over 19 h and (D) the corresponding kinetic profile of HBt (red, tR = 1.2 min), BtC (black, tR = 5.5 min), C120 (blue, tR = 11.6 min) and an unknown decomposition product (green, tR = 2.4 min). (TIFF) [file pone.0053874.s002.tif]

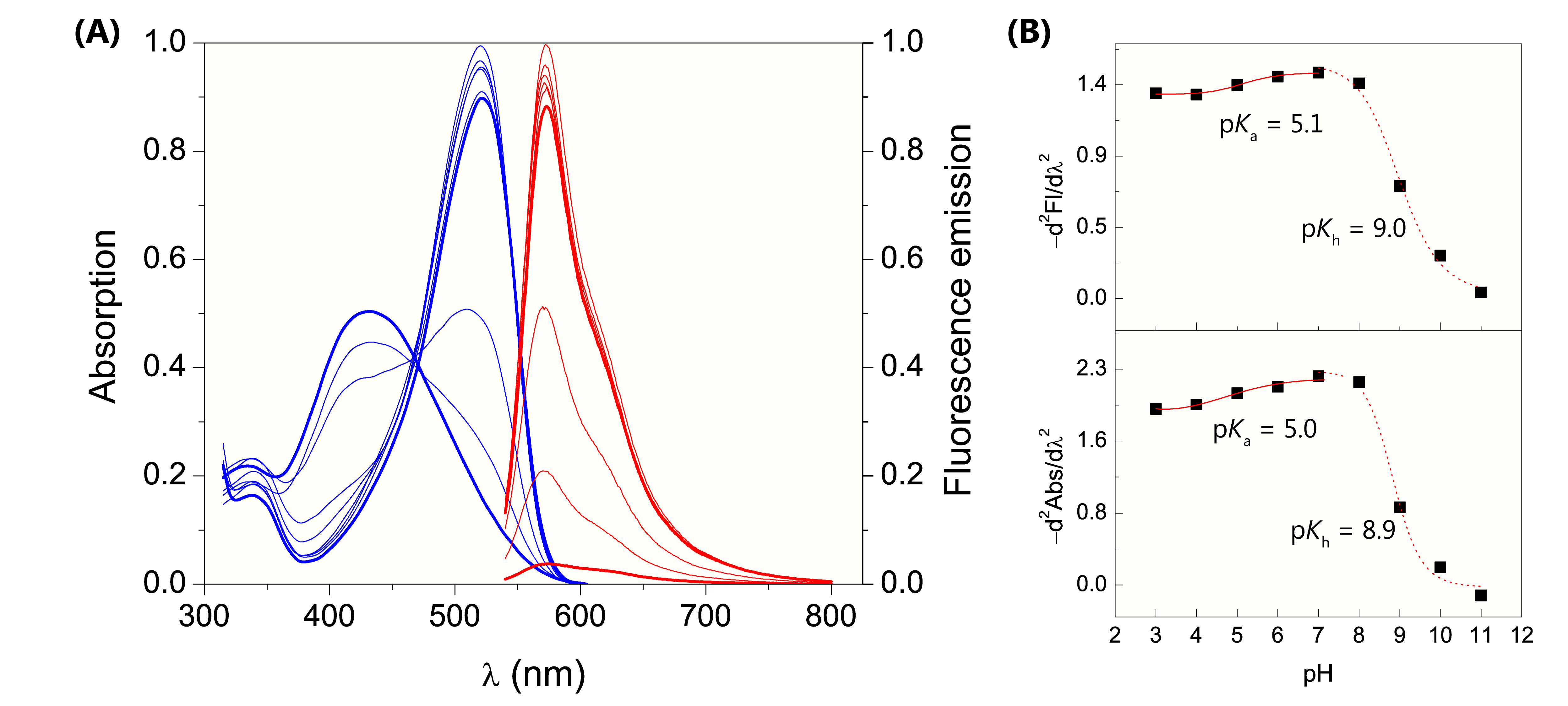

Supplement: Figure S3 — (A) Effect of pH on the absorption and fluorescence spectra of BtC, and (B) dependence of the second-order absorption and emission maxima on the pH. (TIFF) [file pone.0053874.s003.tif]

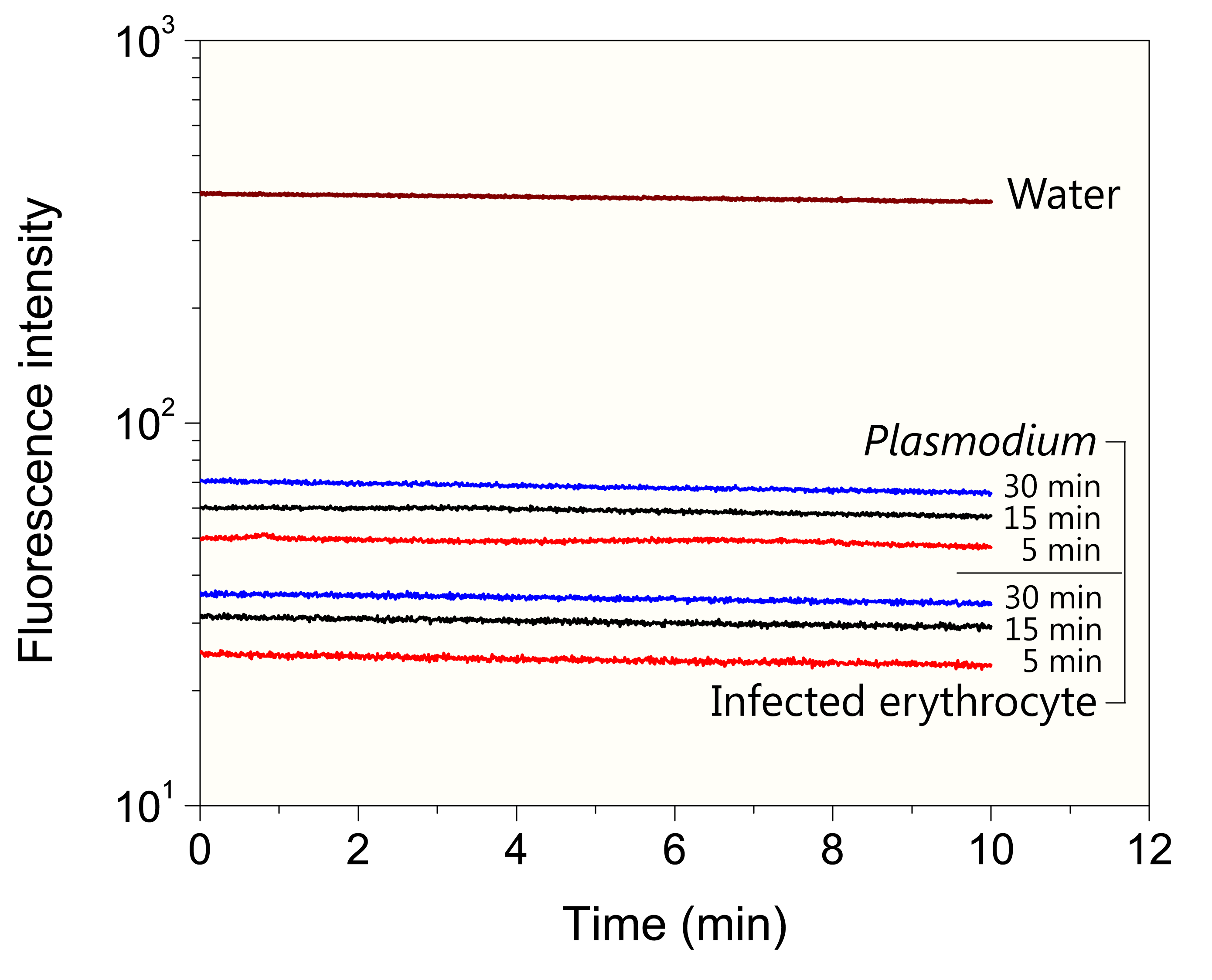

Supplement: Figure S4 — Fluorescence decay of BtC in water, in isolated P. chabaudi and in P. chabaudi -infected red blood cells. Experiments with parasites were performed with an incubation – centrifugation – washing – resuspension sequence. [BtC] = 50 µmol L–1; EM 570 nm. (TIFF) [file pone.0053874.s004.tif]

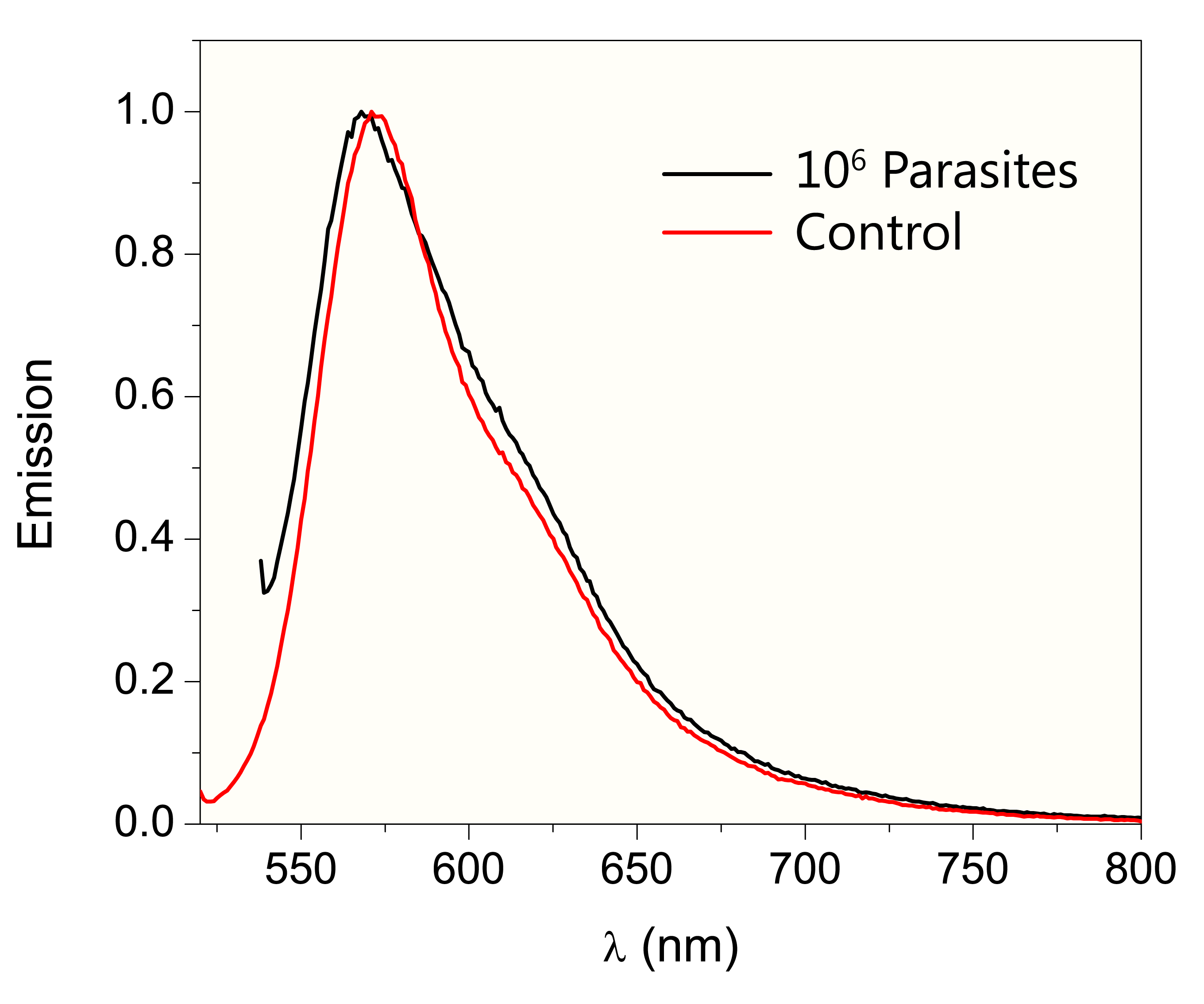

Supplement: Figure S5 — Fluorescence profiles of BtC in aqueous solution (control) and in suspension containing P. chabaudi -infected erythrocytes ([BtC] = 50 µmol L–1, 5 min incubation and washing (5×), EX 520 nm). (TIFF) [file pone.0053874.s005.tif]

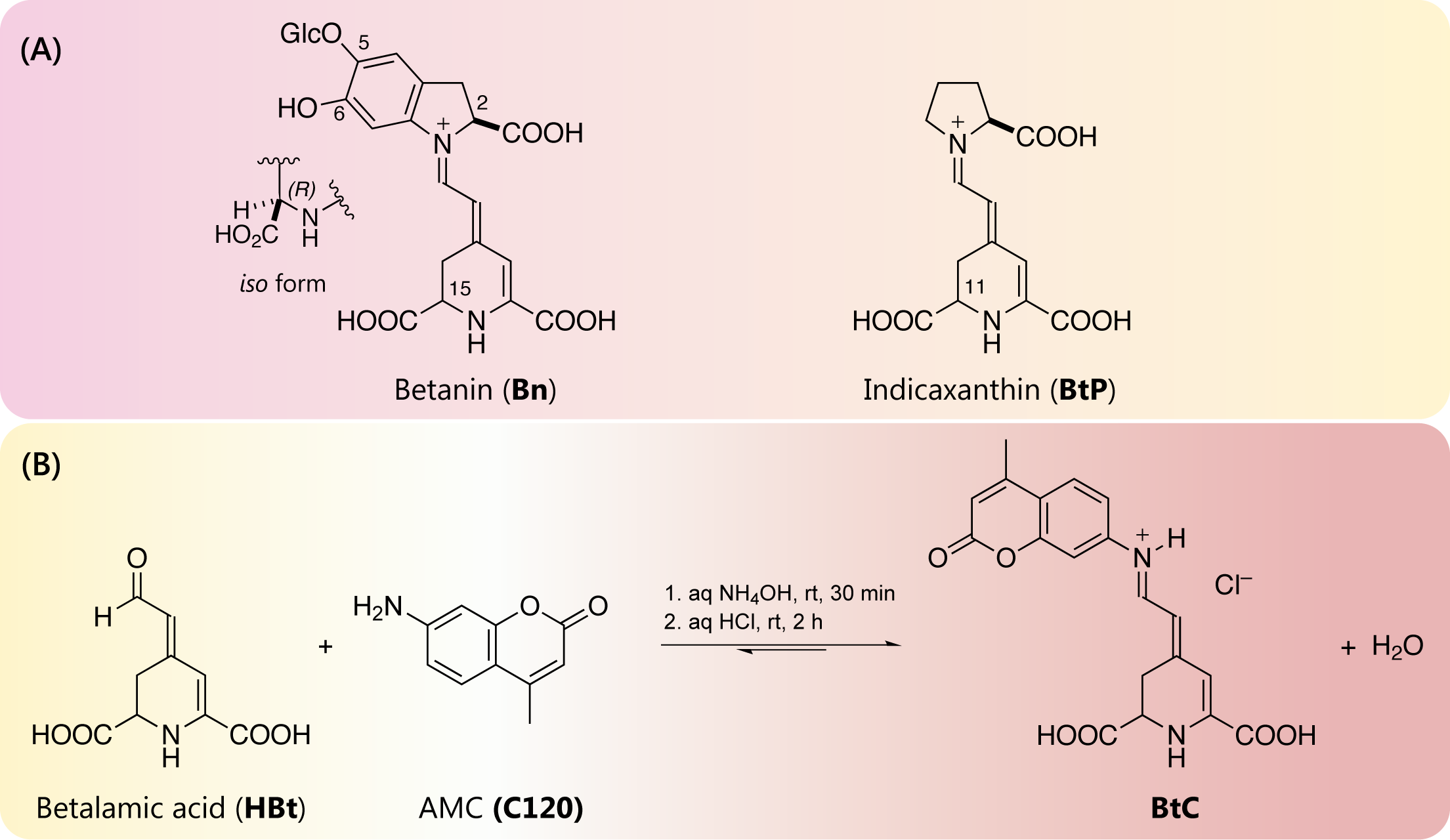

Supplement: Scheme S1 — (A) Chemical structures of betanin (Bn) and indicaxanthin (BtP); (B) semisynthesis of BtC. (TIFF) [file pone.0053874.s007.tif]

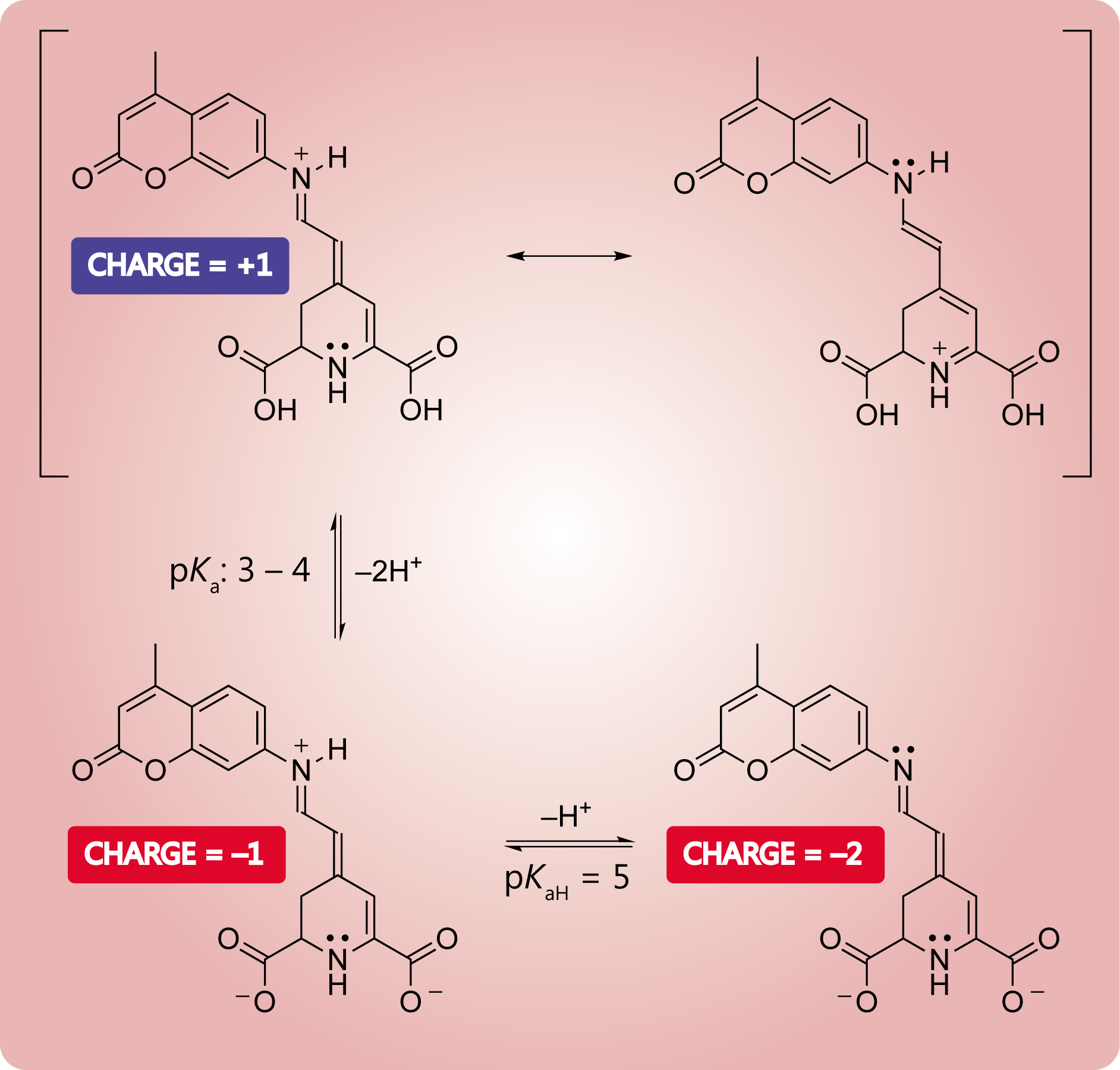

Supplement: Scheme S2 — Acid–base equilibria of BtC. (TIFF) [file pone.0053874.s008.tif]
