# Supplementary material for: A Nature-Inspired Betalainic Probe for Live-Cell Imaging of Plasmodium-Infected Erythrocytes
Source: PLoS One. 2013 Jan 16;8(1):e53874. doi: 10.1371/journal.pone.0053874 (PMC3547039; doi:10.1371/journal.pone.0053874)
Supplement: File S1 — NMR and HRMS spectra of BtP and BtC and cartesian coordinates of optimized structures. (DOCX) [file pone.0053874.s006.docx]

**SUPPORTING INFORMATION**

**A nature-inspired betalainic probe for live-cell imaging of *Plasmodium*-infected erythrocytes**

Letícia Christina Pires Gonçalves, Renata Rosito Tonelli, Piero Bagnaresi,
Renato Arruda Mortara, Antonio Gilberto Ferreira and Erick Leite Bastos

**SPECTRA**

*Indicaxanthin (****BtP****)*

Signals with a brown background correspond to impurities (< 5%).

**HRMS** (*m/z*): [M + H]^+^ calculated for C_14_H_17_N_2_O_6_^+^: 309.1087; observed: 309.1082 (dif.: 1.62 ppm).

***BtC***

**HRMS** (*m/z*): [M + H]^+^ calculated for C_19_H_17_N_2_O_6_^+^, 369.1078; observed, 369.1082 (dif.: –1.08 ppm).

**COORDINATES**

**BtC**, fully protonated

| C | 0.156121000 | -1.178448000 | -0.127763000 |
| --- | --- | --- | --- |
| C | 1.447021000 | -1.645367000 | -0.307526000 |
| C | 2.593190000 | -0.856577000 | -0.175320000 |
| C | 2.540498000 | 0.589061000 | 0.275200000 |
| C | 3.866164000 | -1.422864000 | -0.340424000 |
| C | 3.738808000 | 1.368302000 | -0.266509000 |
| C | 5.004650000 | -0.658309000 | -0.127803000 |
| N | 4.968251000 | 0.657521000 | 0.008736000 |
| C | 3.866806000 | 2.778109000 | 0.284123000 |
| C | 6.392601000 | -1.241660000 | -0.021698000 |
| O | 2.706090000 | 3.433388000 | 0.225548000 |
| O | 4.902994000 | 3.237301000 | 0.688530000 |
| O | 6.409198000 | -2.561116000 | -0.180093000 |
| O | 7.352854000 | -0.546206000 | 0.188339000 |
| H | -0.034736000 | -0.135009000 | 0.096430000 |
| H | 1.586358000 | -2.692767000 | -0.562610000 |
| H | 1.633291000 | 1.098563000 | -0.046402000 |
| H | 2.547374000 | 0.600727000 | 1.373182000 |
| H | 3.982331000 | -2.479615000 | -0.539356000 |
| H | 3.627469000 | 1.462853000 | -1.358899000 |
| H | 5.822627000 | 1.177944000 | 0.203187000 |
| H | 2.839845000 | 4.343902000 | 0.541453000 |
| H | 7.326631000 | -2.875915000 | -0.105732000 |
| C | -2.274764000 | -1.512972000 | -0.088382000 |
| C | -2.634673000 | -0.200137000 | -0.352001000 |
| C | -3.235782000 | -2.456069000 | 0.287445000 |
| C | -3.966537000 | 0.172316000 | -0.203576000 |
| H | -1.935039000 | 0.549182000 | -0.701513000 |
| C | -4.557202000 | -2.066994000 | 0.412368000 |
| H | -2.948060000 | -3.482006000 | 0.493821000 |
| C | -4.955302000 | -0.744745000 | 0.177085000 |
| O | -4.250202000 | 1.467571000 | -0.466525000 |
| H | -5.296009000 | -2.803194000 | 0.706099000 |
| C | -6.329605000 | -0.267104000 | 0.290552000 |
| C | -5.542775000 | 1.977632000 | -0.371321000 |
| C | -6.577949000 | 1.030739000 | 0.023446000 |
| C | -7.420454000 | -1.212304000 | 0.694234000 |
| O | -5.698975000 | 3.141518000 | -0.616895000 |
| H | -7.574926000 | 1.449486000 | 0.088327000 |
| H | -7.505826000 | -2.040807000 | -0.015971000 |
| H | -7.223178000 | -1.642157000 | 1.681340000 |
| H | -8.381846000 | -0.699625000 | 0.733713000 |
| N | -0.927397000 | -1.941606000 | -0.200128000 |
| H | -0.792841000 | -2.938268000 | -0.331330000 |

**BtC** carboxylate 1

| C | 0.172298000 | -1.114413000 | -0.129631000 |
| --- | --- | --- | --- |
| C | 1.432169000 | -1.607976000 | -0.275566000 |
| C | 2.615572000 | -0.815260000 | -0.191792000 |
| C | 2.564664000 | 0.659279000 | 0.135490000 |
| C | 3.853785000 | -1.410256000 | -0.297433000 |
| C | 3.765326000 | 1.403582000 | -0.416282000 |
| C | 5.026913000 | -0.628215000 | -0.136495000 |
| N | 4.991149000 | 0.667295000 | -0.175324000 |
| C | 3.987930000 | 2.840891000 | 0.196142000 |
| C | 6.387288000 | -1.232969000 | 0.097073000 |
| O | 2.948259000 | 3.457225000 | 0.452685000 |
| O | 5.201612000 | 3.125748000 | 0.310723000 |
| O | 6.363056000 | -2.571198000 | 0.066139000 |
| O | 7.377608000 | -0.575444000 | 0.282728000 |
| H | 0.003796000 | -0.056847000 | 0.032514000 |
| H | 1.559886000 | -2.674152000 | -0.451837000 |
| H | 1.673874000 | 1.150828000 | -0.251527000 |
| H | 2.518729000 | 0.773837000 | 1.227326000 |
| H | 3.960256000 | -2.480765000 | -0.410899000 |
| H | 3.673046000 | 1.531043000 | -1.504503000 |
| H | 5.781231000 | 1.310608000 | 0.018511000 |
| H | 7.269563000 | -2.887052000 | 0.214912000 |
| C | -2.275796000 | -1.456438000 | -0.072899000 |
| C | -2.644094000 | -0.123851000 | -0.210544000 |
| C | -3.259420000 | -2.433841000 | 0.146862000 |
| C | -3.988697000 | 0.218402000 | -0.108973000 |
| H | -1.935605000 | 0.669649000 | -0.411477000 |
| C | -4.587786000 | -2.072079000 | 0.235286000 |
| H | -2.971774000 | -3.474890000 | 0.256835000 |
| C | -4.990182000 | -0.734590000 | 0.114466000 |
| O | -4.277792000 | 1.535769000 | -0.253233000 |
| H | -5.331900000 | -2.841091000 | 0.408000000 |
| C | -6.368672000 | -0.277983000 | 0.199690000 |
| C | -5.573999000 | 2.022551000 | -0.188223000 |
| C | -6.619969000 | 1.039301000 | 0.053660000 |
| C | -7.471508000 | -1.264348000 | 0.444671000 |
| O | -5.737163000 | 3.205561000 | -0.334772000 |
| H | -7.623566000 | 1.443337000 | 0.106183000 |
| H | -7.507373000 | -2.017854000 | -0.348450000 |
| H | -7.322973000 | -1.791516000 | 1.392384000 |
| H | -8.439025000 | -0.762296000 | 0.481772000 |
| N | -0.942638000 | -1.881969000 | -0.154716000 |
| H | -0.807325000 | -2.882119000 | -0.216638000 |

**BtC** carboxylate 2

| C | 0.194564000 | -1.185773000 | -0.129025000 |
| --- | --- | --- | --- |
| C | 1.446968000 | -1.678029000 | -0.279776000 |
| C | 2.651664000 | -0.898334000 | -0.208915000 |
| C | 2.600163000 | 0.579514000 | 0.122152000 |
| C | 3.874566000 | -1.491057000 | -0.326157000 |
| C | 3.844629000 | 1.302325000 | -0.398124000 |
| C | 5.062294000 | -0.741031000 | -0.093005000 |
| N | 5.038264000 | 0.559620000 | -0.052923000 |
| C | 3.968957000 | 2.726972000 | 0.101391000 |
| C | 6.468363000 | -1.384115000 | 0.139755000 |
| O | 2.829716000 | 3.410730000 | -0.090396000 |
| O | 4.965142000 | 3.200233000 | 0.582221000 |
| O | 6.474729000 | -2.616007000 | 0.029041000 |
| O | 7.336130000 | -0.525684000 | 0.384396000 |
| H | 0.022516000 | -0.129144000 | 0.033470000 |
| H | 1.577431000 | -2.744276000 | -0.449927000 |
| H | 1.726169000 | 1.066097000 | -0.310362000 |
| H | 2.524334000 | 0.688046000 | 1.212367000 |
| H | 4.004090000 | -2.556769000 | -0.470727000 |
| H | 3.778563000 | 1.358376000 | -1.495818000 |
| H | 5.948756000 | 0.993321000 | 0.153750000 |
| H | 2.972001000 | 4.323134000 | 0.211821000 |
| C | -2.254077000 | -1.510192000 | -0.070694000 |
| C | -2.600421000 | -0.168110000 | -0.183727000 |
| C | -3.262135000 | -2.471277000 | 0.119230000 |
| C | -3.937885000 | 0.198333000 | -0.085676000 |
| H | -1.877212000 | 0.616433000 | -0.363380000 |
| C | -4.583464000 | -2.085273000 | 0.201923000 |
| H | -2.996662000 | -3.520038000 | 0.209082000 |
| C | -4.960756000 | -0.737314000 | 0.106802000 |
| O | -4.197727000 | 1.526245000 | -0.199001000 |
| H | -5.343572000 | -2.843724000 | 0.350361000 |
| C | -6.328928000 | -0.253160000 | 0.188532000 |
| C | -5.483071000 | 2.039162000 | -0.128493000 |
| C | -6.551677000 | 1.073435000 | 0.073865000 |
| C | -7.455433000 | -1.221148000 | 0.396008000 |
| O | -5.615561000 | 3.231230000 | -0.238245000 |
| H | -7.547384000 | 1.496416000 | 0.126273000 |
| H | -7.497206000 | -1.953812000 | -0.416071000 |
| H | -7.328887000 | -1.775075000 | 1.331534000 |
| H | -8.412688000 | -0.699953000 | 0.434820000 |
| N | -0.931587000 | -1.952337000 | -0.144663000 |
| H | -0.802332000 | -2.953554000 | -0.194620000 |

**BtC** dicarboxylate

| C | -0.209784000 | -1.040796000 | 0.088745000 |
| --- | --- | --- | --- |
| C | -1.444924000 | -1.554959000 | 0.230130000 |
| C | -2.678759000 | -0.796025000 | 0.180443000 |
| C | -2.674701000 | 0.687253000 | -0.103850000 |
| C | -3.869523000 | -1.435928000 | 0.287741000 |
| C | -3.921575000 | 1.365183000 | 0.438671000 |
| C | -5.104441000 | -0.718021000 | 0.089479000 |
| N | -5.104582000 | 0.571859000 | 0.120694000 |
| C | -4.117671000 | 2.836014000 | -0.059553000 |
| C | -6.449436000 | -1.462580000 | -0.163528000 |
| O | -3.058075000 | 3.482376000 | -0.171847000 |
| O | -5.309785000 | 3.159580000 | -0.241142000 |
| O | -6.355677000 | -2.700967000 | -0.058852000 |
| O | -7.402349000 | -0.705714000 | -0.411929000 |
| H | -0.049119000 | 0.020486000 | -0.051000000 |
| H | -1.551301000 | -2.629693000 | 0.373764000 |
| H | -1.816538000 | 1.199344000 | 0.330504000 |
| H | -2.601290000 | 0.843352000 | -1.189269000 |
| H | -3.962329000 | -2.508694000 | 0.406309000 |
| H | -3.859652000 | 1.410804000 | 1.535954000 |
| H | -5.971009000 | 1.090059000 | -0.072348000 |
| C | 2.246501000 | -1.418170000 | 0.041182000 |
| C | 2.636215000 | -0.079496000 | 0.098101000 |
| C | 3.240375000 | -2.416127000 | -0.063822000 |
| C | 3.988015000 | 0.236604000 | 0.040216000 |
| H | 1.928363000 | 0.733269000 | 0.194841000 |
| C | 4.572339000 | -2.077152000 | -0.115576000 |
| H | 2.943700000 | -3.459665000 | -0.109666000 |
| C | 4.990410000 | -0.736797000 | -0.067923000 |
| O | 4.294385000 | 1.561422000 | 0.101949000 |
| H | 5.312323000 | -2.865470000 | -0.198838000 |
| C | 6.370345000 | -0.300896000 | -0.120444000 |
| C | 5.594043000 | 2.026938000 | 0.061526000 |
| C | 6.637003000 | 1.022541000 | -0.058193000 |
| C | 7.473151000 | -1.311556000 | -0.242354000 |
| O | 5.768845000 | 3.219490000 | 0.127876000 |
| H | 7.647914000 | 1.409735000 | -0.092737000 |
| H | 7.462324000 | -2.007301000 | 0.602630000 |
| H | 7.361365000 | -1.904619000 | -1.155565000 |
| H | 8.447365000 | -0.821053000 | -0.269611000 |
| N | 0.928387000 | -1.825151000 | 0.086342000 |
| H | 0.776993000 | -2.823210000 | 0.111387000 |

**BtC** charge -2

| C | 0.138920000 | -1.125549000 | 0.016977000 |
| --- | --- | --- | --- |
| C | 1.420817000 | -1.575735000 | -0.226056000 |
| C | 2.616562000 | -0.817412000 | -0.149047000 |
| C | 2.638009000 | 0.615985000 | 0.345569000 |
| C | 3.842541000 | -1.408207000 | -0.410092000 |
| C | 3.835982000 | 1.382099000 | -0.197587000 |
| C | 5.052198000 | -0.706556000 | -0.208054000 |
| N | 5.045177000 | 0.590629000 | -0.034129000 |
| C | 4.026644000 | 2.810157000 | 0.403085000 |
| C | 6.435565000 | -1.418916000 | -0.186884000 |
| O | 2.968464000 | 3.448745000 | 0.589144000 |
| O | 5.217861000 | 3.154456000 | 0.583712000 |
| O | 6.404621000 | -2.637880000 | -0.454760000 |
| O | 7.395967000 | -0.667462000 | 0.084850000 |
| H | -0.003247000 | -0.066243000 | 0.258567000 |
| H | 1.519353000 | -2.627122000 | -0.486417000 |
| H | 1.739871000 | 1.172372000 | 0.082344000 |
| H | 2.675455000 | 0.598737000 | 1.444046000 |
| H | 3.928476000 | -2.458676000 | -0.657558000 |
| H | 3.686785000 | 1.520740000 | -1.281511000 |
| H | 5.919203000 | 1.083569000 | 0.153387000 |
| C | -2.177376000 | -1.469860000 | 0.056320000 |
| C | -2.602318000 | -0.146883000 | -0.242058000 |
| C | -3.206262000 | -2.409101000 | 0.395080000 |
| C | -3.940399000 | 0.188298000 | -0.175635000 |
| H | -1.899330000 | 0.615429000 | -0.554831000 |
| C | -4.524145000 | -2.052055000 | 0.462930000 |
| H | -2.886260000 | -3.423454000 | 0.607837000 |
| C | -4.949400000 | -0.730335000 | 0.180475000 |
| O | -4.268096000 | 1.483618000 | -0.495061000 |
| H | -5.263346000 | -2.799227000 | 0.737032000 |
| C | -6.302883000 | -0.275967000 | 0.214934000 |
| C | -5.558276000 | 1.955746000 | -0.485092000 |
| C | -6.586960000 | 1.018814000 | -0.103548000 |
| C | -7.407983000 | -1.221269000 | 0.599544000 |
| O | -5.737934000 | 3.119005000 | -0.797181000 |
| H | -7.597863000 | 1.407731000 | -0.091673000 |
| H | -7.453461000 | -2.071770000 | -0.088393000 |
| H | -7.244948000 | -1.626156000 | 1.603534000 |
| H | -8.375235000 | -0.714179000 | 0.584784000 |
| N | -0.916595000 | -1.947075000 | 0.013850000 |

**BtC** charge -2, 2

| C | 0.245670000 | -0.840261000 | 0.047376000 |
| --- | --- | --- | --- |
| C | 1.472284000 | -1.356790000 | -0.137963000 |
| C | 2.738406000 | -0.653386000 | -0.096324000 |
| C | 2.839288000 | 0.821197000 | 0.169559000 |
| C | 3.887852000 | -1.345879000 | -0.225709000 |
| C | 4.132497000 | 1.401765000 | -0.408722000 |
| C | 5.192749000 | -0.678908000 | -0.075720000 |
| N | 5.327596000 | 0.593867000 | -0.130047000 |
| C | 4.306394000 | 2.886251000 | 0.068184000 |
| C | 6.398343000 | -1.643345000 | 0.055447000 |
| O | 4.714182000 | 3.691810000 | -0.803621000 |
| O | 3.974190000 | 3.124027000 | 1.256587000 |
| O | 6.263657000 | -2.680842000 | -0.657556000 |
| O | 7.329811000 | -1.339493000 | 0.822105000 |
| H | 0.075162000 | 0.210261000 | 0.240794000 |
| H | 1.547424000 | -2.431589000 | -0.320784000 |
| H | 1.991752000 | 1.368442000 | -0.259989000 |
| H | 2.830193000 | 1.033377000 | 1.247693000 |
| H | 3.912399000 | -2.416505000 | -0.402968000 |
| H | 4.061718000 | 1.452081000 | -1.504812000 |
| C | -2.203198000 | -1.327759000 | 0.042228000 |
| C | -2.658808000 | 0.000504000 | -0.030375000 |
| C | -3.171588000 | -2.368738000 | 0.109226000 |
| C | -4.020456000 | 0.252752000 | -0.025277000 |
| H | -1.976525000 | 0.837277000 | -0.098827000 |
| C | -4.512724000 | -2.088218000 | 0.109657000 |
| H | -2.831124000 | -3.398720000 | 0.164821000 |
| C | -4.990781000 | -0.763091000 | 0.045292000 |
| O | -4.391993000 | 1.565424000 | -0.098777000 |
| H | -5.220860000 | -2.908629000 | 0.163349000 |
| C | -6.378155000 | -0.387665000 | 0.045277000 |
| C | -5.708649000 | 1.970979000 | -0.105372000 |
| C | -6.708141000 | 0.926594000 | -0.026004000 |
| C | -7.444819000 | -1.443108000 | 0.123949000 |
| O | -5.933696000 | 3.159848000 | -0.176634000 |
| H | -7.737091000 | 1.264622000 | -0.029215000 |
| H | -7.372895000 | -2.136846000 | -0.719866000 |
| H | -7.344523000 | -2.033795000 | 1.040162000 |
| H | -8.438582000 | -0.991429000 | 0.113411000 |
| N | -0.891871000 | -1.669887000 | 0.053620000 |
| H | -0.693321000 | -2.659973000 | 0.056029000 |

**BtC** charge -3

| C | 0.179937000 | -0.878154000 | -0.068065000 |
| --- | --- | --- | --- |
| C | 1.440748000 | -1.373482000 | -0.119455000 |
| C | 2.713412000 | -0.669626000 | -0.064237000 |
| C | 2.836693000 | 0.825520000 | 0.098364000 |
| C | 3.870178000 | -1.368707000 | -0.101095000 |
| C | 4.146352000 | 1.340036000 | -0.507505000 |
| C | 5.173690000 | -0.698723000 | 0.050206000 |
| N | 5.330197000 | 0.565357000 | -0.098456000 |
| C | 4.339166000 | 2.866069000 | -0.220103000 |
| C | 6.372909000 | -1.636658000 | 0.315092000 |
| O | 4.734923000 | 3.561489000 | -1.194530000 |
| O | 4.050955000 | 3.263506000 | 0.937058000 |
| O | 6.424205000 | -2.614078000 | -0.486521000 |
| O | 7.140282000 | -1.383372000 | 1.269214000 |
| H | 0.024622000 | 0.199184000 | 0.023184000 |
| H | 1.512621000 | -2.457177000 | -0.210326000 |
| H | 1.999057000 | 1.349917000 | -0.373996000 |
| H | 2.815482000 | 1.104500000 | 1.160687000 |
| H | 3.880927000 | -2.450075000 | -0.197311000 |
| H | 4.095333000 | 1.249319000 | -1.602954000 |
| C | -2.149222000 | -1.331754000 | -0.090618000 |
| C | -2.666229000 | 0.018213000 | 0.024928000 |
| C | -3.163462000 | -2.372747000 | -0.172311000 |
| C | -4.014805000 | 0.252376000 | 0.050049000 |
| H | -1.998704000 | 0.868041000 | 0.093729000 |
| C | -4.495233000 | -2.111497000 | -0.144974000 |
| H | -2.789993000 | -3.388606000 | -0.258315000 |
| C | -5.001834000 | -0.777418000 | -0.031976000 |
| O | -4.425480000 | 1.565187000 | 0.161220000 |
| H | -5.200060000 | -2.937143000 | -0.210885000 |
| C | -6.355741000 | -0.417914000 | 0.001754000 |
| C | -5.751221000 | 1.951153000 | 0.197267000 |
| C | -6.725200000 | 0.912188000 | 0.113190000 |
| C | -7.429388000 | -1.473814000 | -0.083557000 |
| O | -5.973103000 | 3.154130000 | 0.298583000 |
| H | -7.763751000 | 1.219479000 | 0.141673000 |
| H | -7.353097000 | -2.042615000 | -1.016898000 |
| H | -7.345198000 | -2.192907000 | 0.738796000 |
| H | -8.422524000 | -1.018373000 | -0.040149000 |
| N | -0.900692000 | -1.738046000 | -0.131422000 |
